# High Per formance and Flexible Supercapacitors based on Carbonized Bamboo Fibers for Wide Temperature Applications

**DOI:** 10.1038/srep31704

**Published:** 2016-08-22

**Authors:** Camila Zequine, C. K. Ranaweera, Z. Wang, Sweta Singh, Prashant Tripathi, O. N. Srivastava, Bipin Kumar Gupta, K. Ramasamy, P. K. Kahol, P. R. Dvornic, Ram K. Gupta

**Affiliations:** 1 Department of Chemistry, Pittsburg State University, 1701 S. Broadway, Pittsburg, KS 66762, USA; 2 Department of Physics, Banaras Hindu University, Varanasi, Uttar Pradesh, 221004, India; 3 CSIR -National Physical Laboratory, Dr. K.S. Krishnan Road, New Delhi 110012, India; 4 Center for Integrated Nanotechnologies, Los Alamos National Laboratory, Albuquerque, NM 87545, USA; 5 Department of Physics, Pittsburg State University, 1701 S. Broadway, Pittsburg, KS 66762, USA

## Abstract

High performance carbonized bamboo fibers were synthesized for a wide range of temperature dependent energy storage applications. The structural and electrochemical properties of the carbonized bamboo fibers were studied for flexible supercapacitor applications. The galvanostatic charge-discharge studies on carbonized fibers exhibited specific capacity of ~510F/g at 0.4 A/g with energy density of 54 Wh/kg. Interestingly, the carbonized bamboo fibers displayed excellent charge storage stability without any appreciable degradation in charge storage capacity over 5,000 charge-discharge cycles. The symmetrical supercapacitor device fabricated using these carbonized bamboo fibers exhibited an areal capacitance of ~1.55 F/cm^2^ at room temperature. In addition to high charge storage capacity and cyclic stability, the device showed excellent flexibility without any degradation to charge storage capacity on bending the electrode. The performance of the supercapacitor device exhibited ~65% improvement at 70 °C compare to that at 10 °C. Our studies suggest that carbonized bamboo fibers are promising candidates for stable, high performance and flexible supercapacitor devices.

Batteries and capacitors are widely utilized energy storage devices^1^. The energy storage and delivery mechanisms in these devices distinguish their performances. In batteries, the chemical energy stored is released as electrical energy during the discharge process. On the other hand, capacitors store electrical energy through electrostatic attraction, which makes the amenable for devices that demand high power density^2^. Carbonaceous materials are broadly investigated as capacitor electrodes because of their electrochemical stability and abundancy^3^. There has been growing research interest in utilizing bio-waste materials for capacitor electrodes as they are predominantly composed of carbon^4^,^5^,^6^,^7^,^8^,^9^,^10^,^11^. The use of bio-waste for energy applications is a proficient method to create alternate energy resources and simultaneously ensure efficient waste management^7^,^12^. The bio-waste materials are specifically rich in carbon and also are a good source for large surface area and porous materials for use as electrodes^3^,^13^,^14^,^15^.

Guo *et al.* used hierarchical porous carbon from sulfonated pitch and studied the effect of the KOH activation on the porosity and the specific surface area^16^. A maximum specific surface area of 3548 m^2^/g was obtained with a KOH to sulfonated pitch ratio of three. A maximum specific capacitance of 263 F/g at a current density of 50 mA/g was observed for these porous carbon obtained after surface activation of the carbonized pitch. Carbonized potato starch was also used for supercapacitor applications after surface activation using KOH^8^. Nitrogen adsorption isothermal experiment suggested that the carbonized potato starch was composed of mainly microporous structure. Electrochemical investigation on carbonized potato starch provided the highest specific capacitance of 335 F/g at current density of 50 mA/g. Xie *et al.* have used KOH activated corn straw and soy protein based carbon for high energy density supercapacitor^2^. The activated carbon demonstrated high single electrode gravimetric and volumetric specific capacitances of 379 F/g and 258 F/cm^3^ at current density of 0.05 A/g with capacitance retention of about 66%.

Recently, bamboo-derived carbon has been attracting considerable attention due to the wide availability of the bamboo tree across the world, and highly porous microstructure of the bamboo fibers^17^,^18^. Yang *et al.* have used carbonized bamboo after surface activation using KOH for energy storage applications^17^. They observed the specific capacitance of ~258 F/g at 0.1 A/g, energy density of ~3.25 Wh/kg and 2.25 kW/kg power density in 6 M KOH electrolyte. Gu *et al.* have utilized microporous bamboo biochars for lithium-sulfur batteries^14^. The bamboo biochars delivered a high initial capacitance of 1295 mAh/g at a low discharge rate of 160 mA/g and high capacitance retention of 550 mAh/g after 150 cycles at a high discharge rate of 800 mA/g with ≥95% coulombic efficiency. The nanocomposites of MnO_2_ decorated bamboo-based activated carbon was synthesized for supercapacitor application and the electrochemical studies showed specific capacitance of ~220 F/g (at 1 A/g) with ~89% capacitance retention after 1,000 cycles^18^. Nevertheless, the specific capacitance of the bamboo-derived carbon electrodes reported in the literature is significantly low for majority of applications. Moreover, to the best of our knowledge there is no report on fabrication of a bamboo-derived carbon supercapacitor device. In this work, we report the carbonized bamboo fibers based supercapacitor electrode exhibiting a high specific capacitance of about 510 F/g at 0.4 A/g with energy density of 54 Wh/kg. The electrode displayed about 100% charge retention over 5,000 charge-discharge cycles. The symmetrical supercapacitor device fabricated by sandwiching carbonized bamboo fiber electrodes showed areal capacitance of ~1.55 F/cm^2^ with an excellent flexibility. In addition, the devices displayed over 65% improvement in charge storage capacity on increasing the temperature from 10 to 70 °C.

## Experimental Details

### Materials

Bamboo fibers were donated from International Fiber Corporation, USA for research and educational activities. N-ethyl-2-pyrrolidone (NMP) and KOH pellets were purchased from MTI Corporation and Fisher Sci, respectively. Ni foam and ion transporting layer (Celgard, 25 μm thick, 39% porosity) were received from MTI Corporation.

### Synthesis of Activated Carbonized Bamboo Fibers

Bamboo fibers were carbonized in two steps. In first step, bamboo fibers were hydrothermally treated. For this, 1 g of bamboo fibers were dispersed in 30 ml 1 M H_2_SO_4_ solution and transferred to 45 ml hydrothermal Teflon container. The hydrothermal reactor was sealed and kept in the autoclave for pretreatment at 180 °C for 24 hrs. After cooling to room temperature naturally, the fibers were filtered and washed several times with DI water. The fibers were dried at 70 °C overnight. In second step, the pretreated bamboo fibers were chemically activated using KOH (1:1 ratio of bamboo fibers and KOH). The ground mixture was heated at 800 °C for 1 h in Argon atmosphere. After cooling to room temperature, the product was washed with 1 M HCl solution followed by DI water. Finally, the product was dried in an oven at 70 °C for 12 hr.

### Structural Characterization

The structural characterization of the carbonized bamboo fibers was performed using X-ray diffraction (XRD), Raman spectroscopy, scanning electron microscopy (SEM) and transmission electron microscope (TEM). The XRD spectra of the sample was taken using Shimadzu X-ray diffractometer using the 2*θ−θ* scan with CuK_α1_ (λ = 1.5406 Å) radiation. Raman studies were carried out using an argon ion laser with a wavelength of 514.5 nm as the excitation source (Model Innova 70, Coherent). The surface morphology of the carbonized bamboo fiber was characterized by scanning electron microscopy (SEM; QUANTA-200). The surface morphology was carried out by using a high resolution transmission electron microscope (HRTEM/TEM, model no. Technai G20-twin, 200 kV with super twin lenses having point and line resolution of 0.144 nm and 0.232 nm, respectively). The surface area was determined by the Brunauer-Emmett-Teller (BET) adsorption method (Micrometrics, USA, ASAP 2020 Models). The bamboo sample was firstly degassed for 24 hours at a holding temperature of 90 °C after that the analysis for nitrogen adsorption was done at liquid nitrogen temperature (−196 °C).

### Electrochemical Characterization

**Figure 1:**
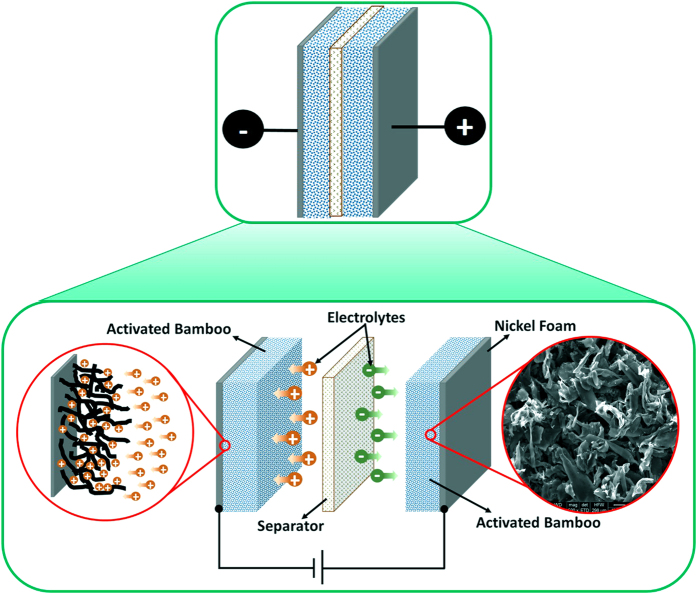
Schematic diagram of supercapacitor device and its individual components.

Electrochemical characterizations of the carbonized bamboo fibers and supercapacitor device were performed using three electrodes and two electrodes technique, respectively. For three electrode measurements, a platinum wire (as a counter electrode), saturated calomel electrode (as a reference electrode) and carbonized bamboo fibers on nickel foam (as a working electrode) were used. The working electrode was prepared by mixing 80 wt.% of the carbonized bamboo fibers, 10 wt.% of acetylene black and 10 wt.% of polyvinylidene difluoride (PVdF) in the presence of N-methyl pyrrolidinone (NMP). After mixing the components, the slurry was pasted onto nickel foam and dried at 60 °C under vacuum for 10 h. The loading mass was accurately measured by weighing the nickel foam before and after electrode preparation using an analytical balance (model MS105DU, Mettler Toledo, max. 120 g, 0.01 mg of resolution). All the electrochemical measurements were performed in an aqueous solution of 3 M KOH, NaOH and LiOH. The supercapacitor device was prepared using two working electrodes separated by ion transporting layer (Fig. 1). The size of the device was 0.25 cm^2^. Before assembling the device, both working electrodes and ion transporting layer were soaked in the 3 M KOH for 1 hr. The charge storage capacity of the electrode and device was studied using cyclic voltammetry (CV) and galvanostatic charge-discharge methods. Electrochemical impedance spectroscopy (EIS) measurements were carried out by applying an AC voltage with 10 mV amplitude in a frequency range from 0.05 Hz to 10 kHz at open circuit potential. Electrochemical measurements were performed on a VersaSTAT 4–500 electrochemical workstation (Princeton Applied Research, USA).

## Results and Discussion

**Figure 2:**
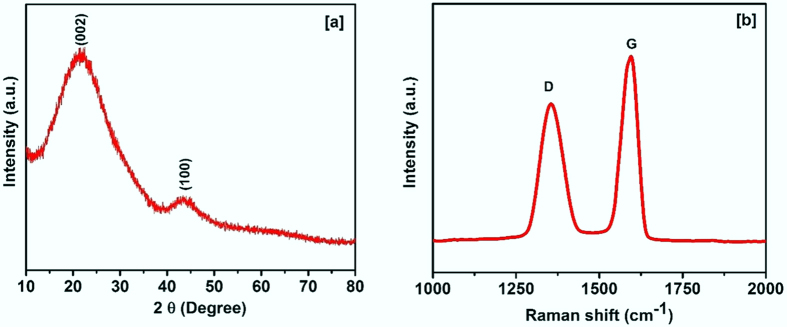
(**a**) XRD patterns and (**b**) Raman spectrum of the carbonized bamboo fibers.

The carbonized bamboo fibers were structurally and morphologically characterized using X-ray diffraction, Raman spectroscopy, scanning electron microscope and transmission electron microscope. Figure 2a shows the XRD pattern of the carbonized bamboo fibers. It is evident from the peak positions (2θ = 21.7 and 43.8 degree) in the XRD pattern that the carbonized bamboo fibers resemble graphitic phase of the carbon. As seen in the XRD patterns, the higher intensity peak was observed at low-angle suggesting the presence of high density micropores in carbonized bamboo fibers^19^. The specific feature of the carbonized bamboo fibers was further analyzed using Raman spectroscopy. Figure 2b shows the Raman spectrum of the carbonized bamboo fibers. The peaks around 1352 cm^−1^ and 1591 cm^−1^ correspond to D and G bands of carbon, respectively. The ratio of D and G band intensity indicates the disorderness in the material. The D/G band intensity of the carbonized bamboo fibers was determined to be 0.74. The higher G intensity compared to D band intensity suggests carbonized bamboo fibers are rich in graphitic phase. It is worth noting that the graphitic phase is the conducting form of the carbon whereas the diamond phase is non-conducting. The presence of high percentage of conducting phase carbon in our sample would be beneficial for reducing the series resistance during the charging and discharging processes.

**Figure 3:**
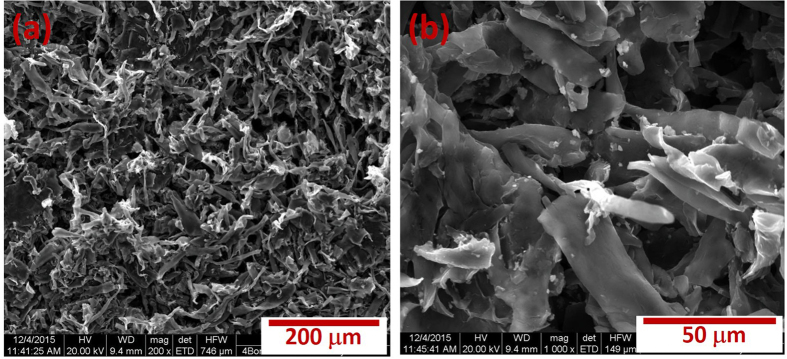
(**a,b**) SEM images of the carbonized bamboo fibers at different magnifications.

The porous structure of bamboo fibers was confirmed by nitrogen adsorption/desorption isotherm measurements (Figure 1S). A typical type IV isotherm characteristic was observed in our sample with a distinct adsorption/desorption hysteresis loop indicating the co-existence of large micropores with some mesopores. The BET surface area was evaluated to be 1120 m^2^/g with pore volume of 0.34 cm^3^/g. In addition, bamboo fibers had an average pore size of 1.22 nm. As a result, carbonized bamboo fibers provide a favorable porous structure for facilitating electron and ion transport in the electrode. Such high surface area and porosity could be very useful for supercapacitors particularly for electrochemical double layer based capacitors. The microstructure of the carbonized bamboo fibers was further studied using scanning electron microscope. The SEM images of the carbonized bamboo fibers at various magnifications are shown in Fig. 3(a,b). As seen in the SEM images, the carbonized bamboo fibers are made of porous structures with average diameter of about 10 μm and several hundred micrometers in length. The porous structure could originate due to activation of the bamboo fibers using KOH as shown in the reaction below^9^:


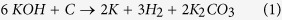


**Figure 4:**
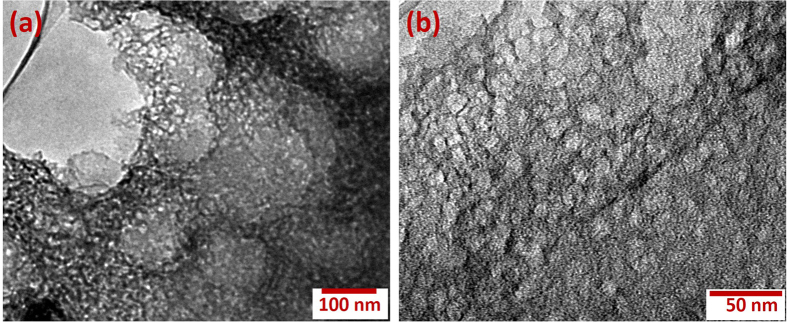
(**a,b**) HR-TEM images of the carbonized bamboo.

The morphology of the carbonized bamboo was further studied using transmission electron microscopy (TEM). As seen in Fig. 4(a,b), the carbonized bamboo shows a three-dimensional, interconnected, random morphology. The TEM images further highlight the porous structure of the carbonized bamboo which is visualized from the low transparency and the alternate light spots throughout the sample (Fig. 4b).

**Figure 5:**
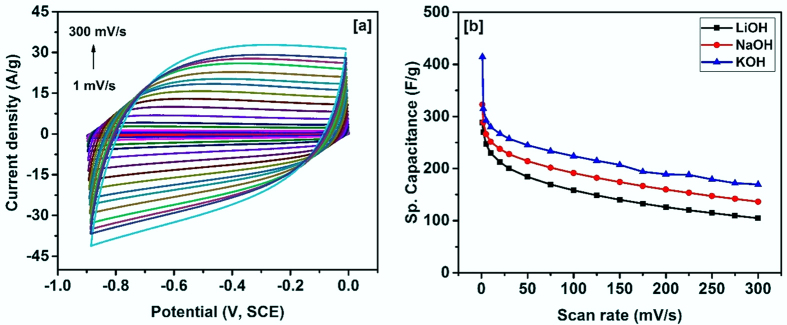
(**a**) CV curves at various scan rates in 3 M KOH and (**b**) variation of specific capacitance versus scan rate for carbonized bamboo fibers in different electrolytes.

In the following sections, we present and discuss the results obtained from the electrochemical studies on carbonized bamboo. Figure 5a shows the CV curves of the carbonized bamboo fibers performed at various scan rates in 3 M KOH electrolyte. As seen in the voltammogram, the shape of the CV curves is identical even at higher scan rates confirming the high electrochemical stability of the electrodes. The near rectangular shape without presence of any redox waves in the CV curves confirms the electrochemical double layer (EDL) mechanism. In such a charge storage process, the electrolyte ions reversibly adsorb and desorb at the surface of the porous carbon materials. Similar CV curves were observed for KOH activated raw rice brans^7^ and from other biomass derived materials^9^,^13^,^16^,^20^,^21^,^22^. Specific capacitance of the carbonized bamboo fibers from CV measurements was calculated using the following expression^23^


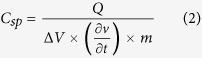


Where, *Q* is the area under the CV curve, ∂*v/*∂*t* is thescan rate, Δ*V* is the potential window and *m* is the mass of the carbonized bamboo fibers. Figure 5b, shows the variation of specific capacitance with scan rates different in each alkaline electrolytes. As seen in the figure, the specific capacitance of the carbonized bamboo fibers decreases with increase in the scan rate. The lower specific capacitance at higher scan rate is due to insufficient time for the electrolyte to adsorb and desorb on the electrode surface^24^. The carbonized bamboo fibers exhibited the maximum specific capacitance of 415 F/g in 3 M KOH electrolyte and lowest in LiOH electrolyte (288 F/g). This observation could be due to the difference in ionic size and thus the mobility of the electrolyte used. The ionic radii of the ions decrease from K^+^ to Li^+^ (ionic radius, K^+^ > Na^+^> Li^+^). However, in the aqueous solution, hydrated radius of Li^+^ is the highest and thus is expected to have the lowest mobility compare to Na^+^ and K^+^.

**Figure 6:**
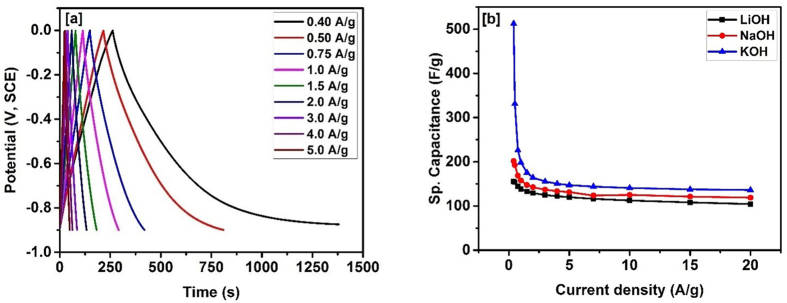
(**a**) Charge-discharge characteristics in 3 M KOH and (**b**)variation of specific capacitance versus applied current for carbonized bamboo fibers in different electrolytes.

The electrochemical properties and charge storage capacity of the carbonized bamboo fibers were further investigated using galvanostatic charge-discharge measurements. Figure 6a, shows the galvanostatic charge-discharge characteristics of the carbonized bamboo fibers in 3 M KOH electrolyte. The galvanostatic charge-discharge time decreases with increase in the current density. The charge-discharge curves were very symmetrical and linear potential-time curves were observed even at higher current densities, suggesting higher stability of the electrode. The specific capacitance (*C*_*sp*_) of the carbonized bamboo fibers from galvanostatic charge-discharge measurements was calculated using the equation given below^23^:


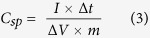


where, *I* is the discharge current (*A*), Δ*t* is the discharge time (s), Δ*V* is the potential window (V), and *m* is the mass (g) of the carbonized bamboo fibers. The effect of applied current and electrolytes on the charge storage capacity of the carbonized bamboo fibers are shown in Fig. 6b. As seen, the highest specific capacitance of 512 F/g at current density of 0.4 A/g was observed in 3 M KOH electrolyte. Specific capacitance was observed to decrease with increasing current density which could be due to insufficient time for the electrolyte ions to diffuse into the inner pores of carbonized bamboo fibers. In addition, it is further observed that the specific capacitance depends on the electrolytes used for the measurements. The highest specific capacitance was observed in KOH electrolyte with the trend of KOH (512 F/g) > NaOH (202 F/g) > LiOH (156 F/g), as observed in the CV measurements too. Table 1 compares some of theimportant parameters reported in literature for activated carbon from bamboo.

In addition to specific capacitance, the energy density and the power density of the carbonized bamboo fibers were calculated using the following expressions^28^:


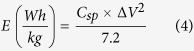



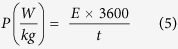


where *C*_*sp*_ (F/g) is the specific capacitance calculated from galvanostatic charge-discharge measurements, Δ*V* (V) is the potential window and *t* (s) is the discharge time. The variations of the specific energy density versus power density (Ragone plot) for carbonized bamboo fibers in three different electrolytes are shown in Figure 2S. As seen, the highest energy density and power density was observed for the KOH electrolyte.

**Figure 7:**
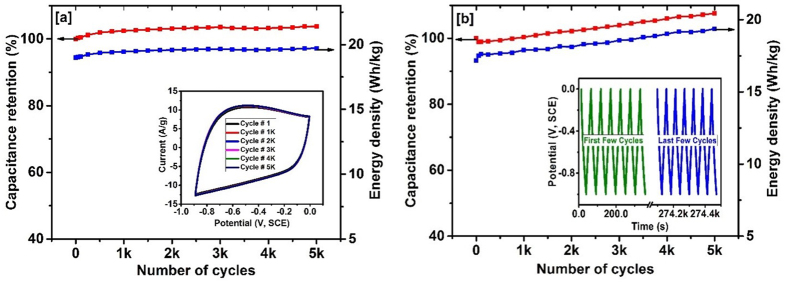
(**a**) % change in the specific capacitance verse number of CV scans, inset figure shows CV curves at various cycles and (**b**) % change in the specific capacitance verse number of charge-discharge cycles in 3 M KOH, inset figure shows potential verses time plot for first and last few cycles.

Further, the stability and flexibility of the carbonized bamboo fibers were investigated for their applications as a flexible and high performance supercapacitor device. Figure 7a, shows the change in specific capacitance and energy density versus number of CV cycles. The CV stability was tested within a potential range of −0.9 to 0 V versus SCE. The shape and the size of the CV curves at various number of CV cycles are given in the inset of Fig. 7a. As seen in the inset figure, the shape and the size of the CV curves are very identical confirming high cyclic stability. The specific capacitance slightly increases initially and then stays almost constant with on increasing CV cycles. In fact, the percentage capacity retention was observed to increase from 100% to 103.8% during the 5,000^th^ cycle of CV scan. The increase in capacitance could be due to the gradual activation of surface and thereby better access of the electrolyte ions into the micropores of the carbonized bamboo fibers with increasing CV cycles. The energy density of the electrode was observed to be constant over 5,000 charge-discharge measurements. The electrochemical stability of the electrode was also tested using galvanostatic charge-discharge technique. Figure 7b, shows the capacitance retention and energy delivery of carbonized bamboo fibers as a function of number of galvanostatic charge-discharge cycles. The specific capacitance and energy density values were observed to be increasing with increasing charge-discharge cycles. The inset of Fig. 7b shows potential versus time curves of the first and last few cycles of galvanostatic charge-discharge measurements. The high performance of the carbonized bamboo is due to its porous structure and high surface area as seen in the TEM images and BET measurement.

**Figure 8:**
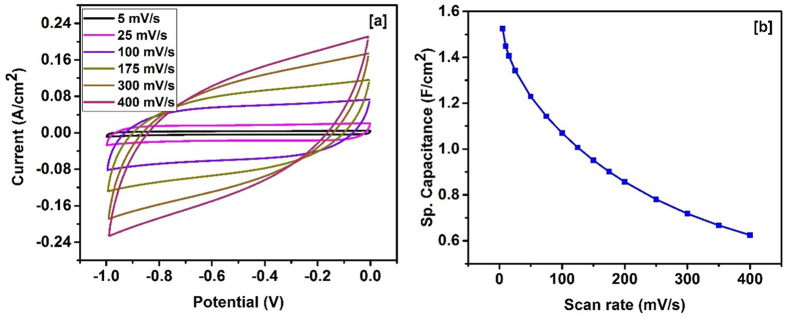
(**a**) CV curves at various scan rates and (**b**) variation of specific capacitance versus scan rate for supercapacitor device.

**Figure 9:**
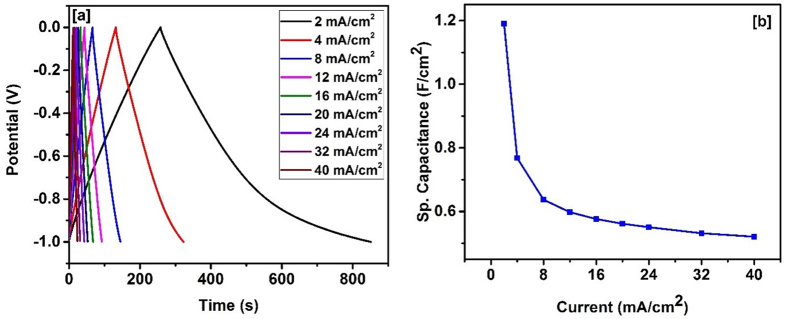
(**a**) Charge-discharge characteristics and (**b**)variation of specific capacitance versus applied current for supercapacitor device.

The potential applications of carbonized bamboo fibers for flexible electronic devices were also investigated. For this, a supercapacitor device was fabricated by sandwiching an ion transporting layer between two carbonized bamboo fiber electrodes. All the electrochemical properties such as cyclic voltammetry and galvanostatic charge-discharge studies of the device were performed in 3 M KOH electrolyte. Figure 8a shows the CV curves of the device at different scan rates. As observed from the curves, the shape of the CV curves is nearly identical even at higher scan rates, indicating high charge transfer stability of the device. The highest areal capacitance of 1.52 F/cm^2^ was observed for the device at scan rate of 5 mV/s (Fig. 8b). The charge storage properties of the device were further investigated using galvanostatic charge-discharge measurements. The potential-time curves (Fig. 9a) are triangular in shape, indicating near ideal capacitive behavior. The variation of specific capacitance versus applied current is shown in Fig. 9b. As seen earlier, the areal capacitance of the bamboo device decreases with increasing current density. An areal capacitance of 1.19 F/cm^2^ was observed at 2 mA/cm^2^ which decreased to 0.52 F/cm^2^ at a discharge current of 40 mA/cm^2^. The flexibility test on the device was performed by measuring CV curves at various bending angles (Fig. 3S). The CV curves at various bending angles were identical and overlapping each other, indicating no change in the capacitance of the device.

**Figure 10:**
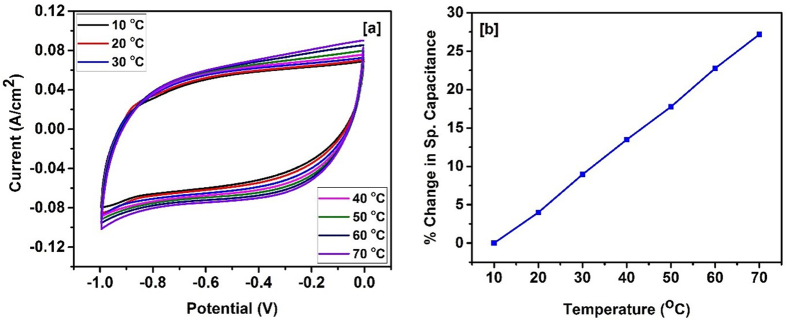
(**a**) CV curves at various temperature and (**b**) % change in specific capacitance versus temperature for the supercapacitor device.

**Figure 11:**
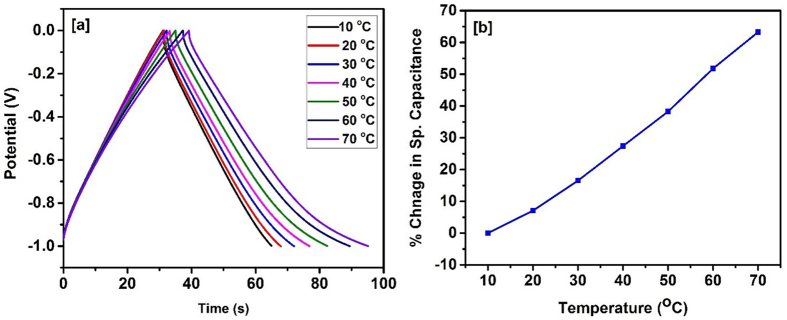
(**a**) Charge discharge characteristics at various temperature and (**b**) % change in specific capacitance versus temperature for the supercapacitor device.

After the stability and flexibility tests, we investigated the electrochemical properties of bamboo derived carbon’s performance at different temperatures. The cyclic voltammetry and galvanostatic charge-discharge measurements were performed at various temperatures (10–70 °C). Figure 10a, shows CV curves of supercapacitor device at different temperatures. As seen, the area of the CV curves increases with increase in temperature suggesting improvement in the charge storage capacity. The shape of the CV curves is similar at all temperatures suggesting high stability of the carbonized bamboo fibers at higher temperatures. The percentage change in the specific capacitance of the device as a function of temperature is shown in Fig. 10b. We observed about 27% improvement in the charge storage capacity by increasing temperature to 70 °C from 10 °C. The effect of temperature on charge storage capacity was also studied using galvanostatic charge-discharge measurements at various temperatures. The charge-discharge time increases with increase in temperature, indicating improvement in charge storage capacity (Fig. 11a). We observed about 65% improvement in capacitance by increasing temperature from 10 to 70 °C. The higher capacitance at higher temperature could be due to higher mobility of electrolyte ions and low series resistance of the device.

The effect of temperature on the electrochemical behavior of the supercapacitor was further investigated using electrochemical impedance spectroscopy (EIS). The variation of real and imaginary impedance of the supercapacitor device at various temperature is shown in Figure 4S. The real and imaginary impedance of the device decreases with increasing temperature. The equivalent series resistance (ESR) of the supercapacitor device decreases with increasing temperature. The decrease in ESR has very positive effect on improvement in the capacitance of the supercapacitor device. The decrease in the ESR value could be due to the enhanced mobility of the ions in the electrolyte which increases the conductivity of the electrolytes^29^. Total impedance of the device was observed to be decreasing with increasing temperature and frequency (Figure 4S). This study indicates that the performance of a supercapacitor device fabricated using carbonized bamboo fibers is better at higher temperature.

## Conclusion

High performance and porous carbon was synthesized by carbonization of bamboo fibers. The electrochemical performance of the carbonized bamboo fibers was investigated in detail. The highest specific capacitance of 512 F/g was observed in KOH electrolyte. The carbonized bamboo fibers showed high power and energy densities of 7.9 kW/kg and 54 Wh/kg, respectively. In addition to high power and energy density they show a very stable performance (~100%) over 5,000 charge and recharge cycles. The potential application of the carbonized bamboo fibers for flexible energy storage device was also tested and an areal capacitance of ~1.55 F/cm^2^ was observed. The device showed no degradation in charge storage capacity on bending. The fabricated device showed an improved performance at higher temperature. An improvement of about 65% was observed on increasing the environmental temperature from 10 to 70 °C. Our studies indicate that the bamboo derived carbon fibers are potential materials for high performance and flexible supercapacitor devices that can be operable over wider temperature range.

## Additional Information

**How to cite this article**: Zequine, C. *et al.* High Performance and Flexible Supercapacitors based on Carbonized Bamboo Fibers for Wide Temperature Applications. *Sci. Rep.* **6**, 31704; doi: 10.1038/srep31704 (2016).

## Supplementary Material

Supplementary Information

## Figures and Tables

**Table 1:** Some electrochemical properties of carbonized bamboo.

Method of activation (Bamboo: Activating agent)	Surface Area (m^2^/g)	Max. Capacitance (F/g)	Retention of capacitance at (%, cycle #)	Electro-chemical testing conditions	Electrolyte used	Reference
KOH (1:3)	1293	34	—	1 mA/cm^2^	H_2_SO_4_	25
KOH (1:4)	3061	258	91 (3,000)	100 mA/g	KOH	17
Commercially activated	—	45	100 (1,000)	1 A/g	Na_2_SO_4_	18
Commercially activated bamboo@MnO_2_	—	222	89 (1,000)	1 A/g	Na_2_SO_4_	18
Steam	1,025	60		1 mV/s	Tetraethylammonium tetrafluoroborate	26
KOH (2:1)	2352	268	97 (5,000)	1 A/g	KOH	27
KOH (1:1)	1120	512	103 (5,000)	400 mA/g	KOH	This work
