# Supplementary material for: High Per formance and Flexible Supercapacitors based on Carbonized Bamboo Fibers for Wide Temperature Applications
Source: Sci Rep. 2016 Aug 22;6:31704. doi: 10.1038/srep31704 (PMC4992840; doi:10.1038/srep31704)
Supplement: Supplementary Information [file srep31704-s1.pdf]

Electronic Supplementary Information

High Performance and Flexible Supercapacitors based on Carbonized Bamboo Fibers for Wide Temperature Applications

Camila Zequine[1], C.K. Ranaweera[1], Z. Wang[1], Sweta Singh[2], Prashant Tripathi[2], O.N. Srivastava[2], Bipin Kumar Gupta[3], K. Ramasamy[4], P.K. Kahol[5], P.R. Dvornic[1], Ram K. Gupta[1*]

[1]Department of Chemistry, Pittsburg State University, 1701 S. Broadway, Pittsburg, KS 66762, USA
[2]Department of Physics, Banaras Hindu University, Varanasi, Uttar Pradesh, 221004, India
[3]CSIR -National Physical Laboratory, Dr. K.S. Krishnan Road, New Delhi 110012, India
[4]Center for Integrated Nanotechnologies, Los Alamos National Laboratory, Albuquerque, NM 87545, USA
[5]Department of Physics, Pittsburg State University, 1701 S. Broadway, Pittsburg, KS 66762, USA

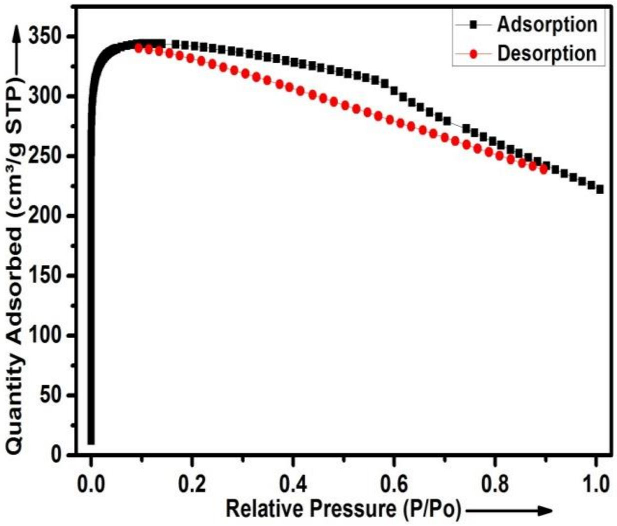

Figure 1S: Nitrogen adsorption/desorption isotherms for bamboo fibers.

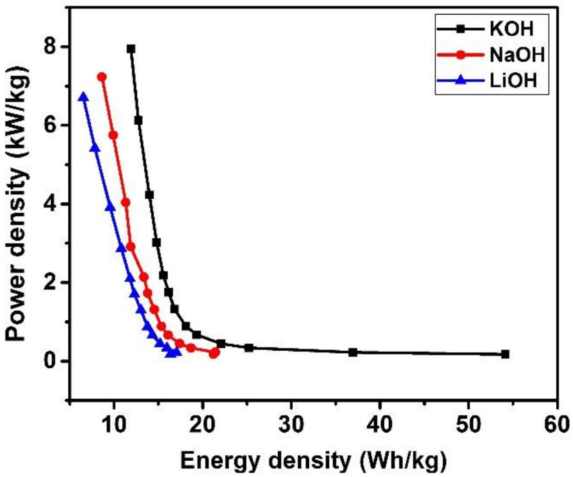

Figure 2S: Ragone plots for bamboo fibers in different electrolytes.

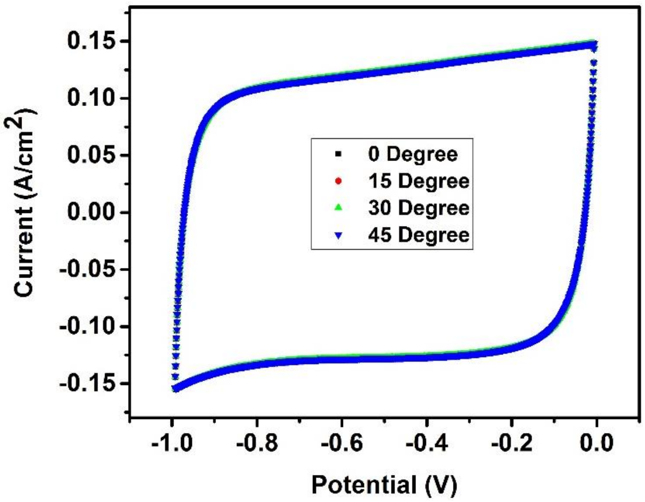

Figure 3S: (a) CV curves of the bamboo device at various bending angles.

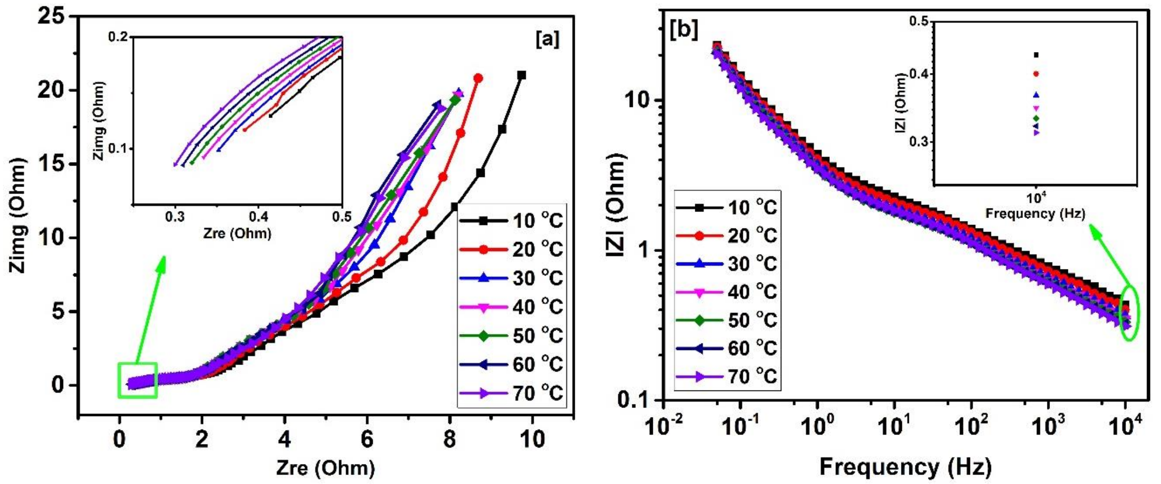

Figure 4S: (a) variation of Z_{re} and I_{img} at various temperatures and (b) variation of IZI as a function of frequency for the supercapacitor device at various temperature.
